# NLRP3: Role in ischemia/reperfusion injuries

**DOI:** 10.3389/fimmu.2022.926895

**Published:** 2022-09-27

**Authors:** Soudeh Ghafouri-Fard, Hamed Shoorei, Yadollah Poornajaf, Bashdar Mahmud Hussen, Yasaman Hajiesmaeili, Atefe Abak, Mohammad Taheri, Ahmad Eghbali

**Affiliations:** ^1^ Department of Medical Genetics, School of Medicine, Shahid Beheshti University of Medical Sciences, Tehran, Iran; ^2^ Clinical Research Development Unit of Tabriz Valiasr Hospital, Tabriz University of Medical Sciences, Tabriz, Iran; ^3^ Department of Anatomical Sciences, Faculty of Medicine, Birjand University of Medical Sciences, Birjand, Iran; ^4^ Faculty of Medicine, Birjand University of Medical Sciences, Birjand, Iran; ^5^ Department of Pharmacognosy, College of Pharmacy, Hawler Medical University, Erbil, Iraq; ^6^ Center of Research and Strategic Studies, Lebanese French University, Erbil, Iraq; ^7^ Faculty of Health, York University, Toronto, ON, Canada; ^8^ Phytochemistry Research Center, Shahid Beheshti University of Medical Sciences, Tehran, Iran; ^9^ Urology and Nephrology Research Center, Shahid Beheshti University of Medical Sciences, Tehran, Iran; ^10^ Institute of Human Genetics, Jena University Hospital, Jena, Germany; ^11^ Anesthesiology Research Center, Mofid Children Hospital, Shahid Beheshti University of Medical Sciences, Tehran, Iran

**Keywords:** NLRP3, ischemia/reperfusion, expression, biomarker, diagnosis

## Abstract

NLR family pyrin domain containing 3 (NLRP3) is expressed in immune cells, especially in dendritic cells and macrophages and acts as a constituent of the inflammasome. This protein acts as a pattern recognition receptor identifying pathogen-associated molecular patterns. In addition to recognition of pathogen-associated molecular patterns, it recognizes damage-associated molecular patterns. Triggering of NLRP3 inflammasome by molecules ATP released from injured cells results in the activation of the inflammatory cytokines IL-1β and IL-18. Abnormal activation of NLRP3 inflammasome has been demonstrated to stimulate inflammatory or metabolic diseases. Thus, NLRP3 is regarded as a proper target for decreasing activity of NLRP3 inflammasome. Recent studies have also shown abnormal activity of NLRP3 in ischemia/reperfusion (I/R) injuries. In the current review, we have focused on the role of this protein in I/R injuries in the gastrointestinal, neurovascular and cardiovascular systems.

## Introduction

*NLR family pyrin domain containing 3* (*NLRP3*) gene is located on chromosome 1q44. The protein encoded by this gene is expressed in immune cells, especially in dendritic cells and macrophages and acts as a constituent of the inflammasome (1). In addition, NLRP3 is expressed in smooth muscle cells, endothelial cells, beta cells and cardiomyocytes (2–4). The pyrin-like protein encoded by this gene has a pyrin domain, a nucleotide-binding site (NBS) domain, and a leucine-rich repeat (LRR) motif. NLRP3 interacts with pyrin domain of apoptosis-associated speck-like protein comprising a CARD. Mutations in this gene have been detected in some organ specific autoimmune disorders. Being an element of the innate immune system, NLRP3 acts as a pattern recognition receptor (PRR) that identifies pathogen-associated molecular patterns (5). PRRs are receptors involved in the recognition of endogenous or exogenous invaders. These receptors can trigger an appropriate immune response to preserve the host integrity. Five groups of PRRs have been identified: Toll-like receptors, nucleotide oligomerization domain-like receptors, retinoic acid-inducible gene-I-like receptors, C-type lectin receptors, and absent in melanoma-2-like receptors (ALRs) (6). Among them, NLRP3 belongs to the NOD-like receptors. NLRP3 in addition to the adaptor ASC protein creates the caspase-1 activating complex NLRP3 inflammasome. In addition to recognition of pathogen-associated molecular patterns (PAMPs), it recognizes Damage-Associated Molecular Patterns (DAMPs).

NLRP3 and some other types of NLRs can create huge cytosolic protein complexes (probably hexamers or heptamers) called inflammasomes, which contribute to the initiation of cleavage and activation of procaspase-1 leading to proteolytic activation of pro- IL-1β and pro-IL-18 (7).

Activation of NLRP3 inflammasome needs a priming step that leads to up-regulation of NLRP3 and IL-1β in addition to NLRP3 post-translational licencing. A succeeding activation step results in the assemblage of the complex and caspase-1-mediated cleavage of pro-IL-18 and pro-IL-1β, permitting their release. The activation step can be triggered by a wide array of factors such as PAMPs and DAMPs, e.g. nigericin toxin, extracellular ATP, silica and cholesterol crystals (8).

In cooperation with the adaptor ASC protein, NLRP3 establishes the caspase-1 activating complex NLRP3 inflammasome. In its inactivate form, cytoplasmic NLRP3 is kept in a complex with HSP90 and SGT1. Crystalline uric acid and extracellular ATP released by injured cells result in the release of HSP90 and SGT1 from the NLRP3 inflammasome and recruitment of ASC protein and caspase-1 to this complex leading to activation of the pro-inflammatory cytokine, IL-1β (5). Consistent with this function, mutations in the *NLRP3* gene have been found to be associated with elevation of IL-1β concentrations in the serum (9, 10). Moreover, incorrect induction of NLRP3 inflammasome has been reported to stimulate inflammatory or metabolic diseases. Thus, NLRP3 is regarded as a suitable target for decreasing activity of NLRP3 inflammasome. Recent studies have also shown abnormal activity of NLRP3 in ischemia/reperfusion (I/R) injuries. I/R injury is the tissue damage resulting from tissue reperfusion with blood after a period of ischemia (11). The lack of blood-born oxygen and nutrients in the course of ischemic period produces a condition in which the reestablishment of circulation leads to inflammation and oxidative damage *via* stimulation of oxidative stress instead of normal function (11). In the current review, we have focused on the role of this protein in I/R injuries in the gastrointestinal, neurovascular and cardiovascular systems.

## Gastrointestinal I/R injury

**Figure 1:**
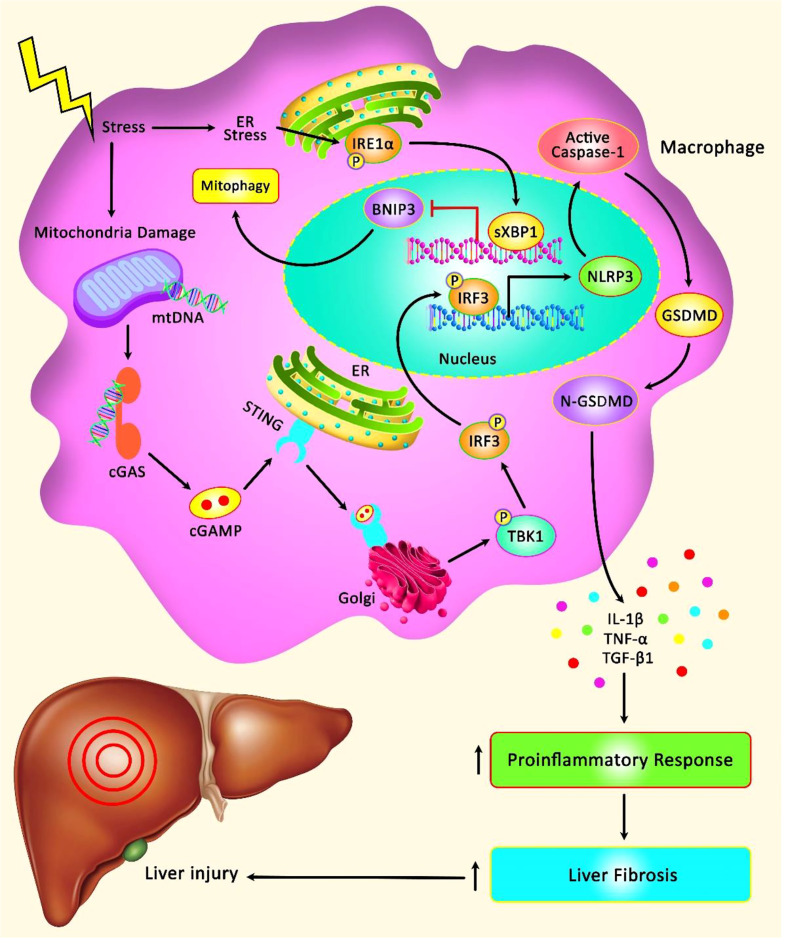
A schematic illustration of the role of NLRP3 Inflammasome involved in the hepatic I/R injury. Mounting evidence has detected that STING/TBK1/NLRP3 signaling cascade can play a remarkable role in modulating innate immune activation and promoting liver IR injury in aged mice. STING can regulate the activation of NLRP3 signaling and excessive secretion of proinflammatory cytokines in the mtDNA-stimulated bone marrow-derived macrophages from aged mice. Moreover, STING upregulation in macrophages can elevate the detrimental role of aging in aggravating liver IR injury and intrahepatic inflammation (12). Furthermore, another research has illustrated that XBP1 can regulate macrophage cGAS/STING/NLRP3 activation *via* elevating macrophage self-mtDNA cytosolic leakage in liver fibrosis. Therefore, macrophage self-mtDNA can play an effective role as an intrinsic trigger for macrophage cGAS/STING activation that can be modulated through regulating XBP1/mitophagy (13).

The role of NLRP3 has been assessed in I/R injury in the liver and intestine (**Figure 1**). NLRP3 inflammasome activation in Kupffer cells can lead to I/R injury in liver cells. The NLRP3-associated hyper-inflammation can be prevented by mitophagy, a process that preserves mitochondrial homeostasis *via* removal of injured mitochondria. An *in vivo* study has shown significant inflammatory responses, over-activation of NLRP3 inflammasome and enhancement of PTEN-induced putative kinase1 (PINK1)-facilitated mitophagy in the process of hepatic I/R. Up-regulation of PINK1 has decreased I/R injury, production of reactive oxygen species (ROS), NLRP3 activation and inflammatory responses in the liver in animal models. *In vitro* anoxia/reoxygenation challenges could trigger NLRP3 activation in Kupffer cells and promote mitophagy. PINK1-mediated enhancement of mitophagy could inhibit NLRP3 activation and reverse the Kupffer cells-mediated inflammatory responses against hepatocytes (14). Another study has found hyper-activation of NLRP3 in both hepatocytes and macrophages of aged animals following I/R. NLRP3 silencing in macrophages has suppressed inflammatory responses and hepatic tissue injury in both young and aged animals. Notably, aged macrophages have exhibited hyper-activation of the STING/TANK- TBK1 signals following I/R. Inhibition of STING could block hyperactivity of NLRP3 signals and abnormal production of inflammatory cytokines in the mtDNA-induced bone marrow-derived macrophages of aged animals. Taken together, STING/NLRP3 axis has been shown to exert critical roles in the induction of inflammatory responses in aged macrophages (15).

Expression of the specific macrophage subunit of vacuolar ATPase (ATP6V0D2) has been reported to be up-regulated in hepatic macrophages after liver I/R surgery. Notably, ATP6V0D2 silencing has led to enhancement of secretion of inflammatory factors and chemokines, and subsequent activation of NLRP3 and exacerbation of hepatic damage. Mechanistically, the intensified activation of NLRP3 has been accompanied by the ATP6V0D2-regulated autophagic flux. ATP6V0D2 silencing has reduced establishment of autophagolysosome and exacerbated hepatic I/R injury *via* nonspecific V-ATPase activation (16).

Another study has shown that SET8 lessens I/R injury in liver *via* suppression of MARK4/NLRP3 inflammasome route (17). Hepatic I/R stimuli have been shown to increase expression of NLRP3 but not ASC. Lower I/R liver injury has been detected in NLRP3(-/-) mice, but not in ASC(-/-) and caspase-1(-/-) mice. NLRP3 knock-out mice has also exhibited decreased inflammatory response, neutrophils infiltration, ROS production, and apoptosis in the liver after I/R. Further functional studies have revealed that NLRP3 regulates chemokine-mediated function and neutrophil recruitment in an independent manner from its function in inflammasomes (18).

NLRP3 inflammasome has also been found to participate in the intestinal I/R injury. Down-regulation of NLRP3, ASC, caspase-1/11, or IL-1β has increased cell survival following intestinal I/R injury. Additionally, intestinal I/R injury has resulted in acute lung injury. The pathological features such as inflammation, ROS production and increased vascular permeability have been ameliorated by NLRP3 down-regulation. Additional studies have shown the critical role of NLRP3 expression in non-bone marrow-derived cells in the evolution of intestinal I/R-induced acute lung injury. In addition, activation of NLRP3 inflammasome in endothelial cells of lung has been shown to contribute to the intestinal I/R-induced acute lung injury (19).

**Figure 2:**
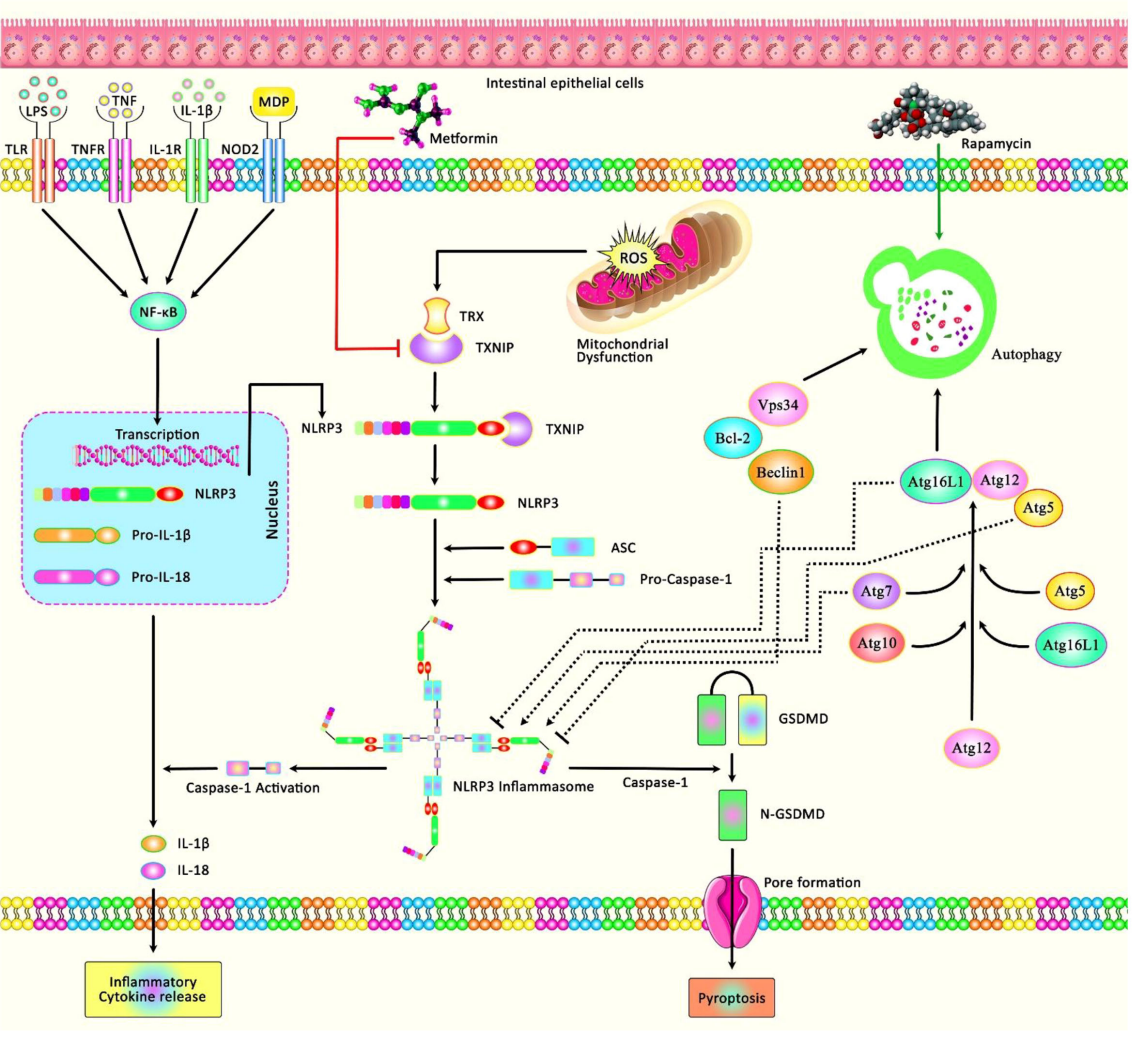
A schematic diagram of the role of NLRP3 involved in I/R Injury in intestine. Mounting evidence has demonstrated that inappropriate activation of NLRP3 could play an effective role in the progression of I/R injury in the intestine by creating an intracellular multi-protein complex known as NLRP3 inflammasome. As an illustration, a recent study has detected that Metformin could protect against intestinal I/R injury and decrease oxidative stress and the inflammatory response *via* downregulating pyroptosis-related proteins, containing NLRP3, active caspase-1, N-GSDMD, and the expression of TXNIP as well as the interaction between TXNIP and NLRP3 (20). Moreover, another research has figured out that Rapamycin through inducing the process of autophagy could attenuate intestinal I/R induced NLRP3 inflammasome activation (21).

I/R injury has also been found to disrupt barrier and induce cell death and pyroptosis. Notably, metformin has been found to protect intestinal barrier against I/R injury, reduce oxidative stress and the inflammatory responses, and decrease expression of NLRP3, cleaved caspase-1, and the N-terminus of GSDMD. In fact, the protective effect of metformin is exerted through modulation of TXNIP/NLRP3/GSDMD proteins (20) (**Figure 2**). Moreover, the autophagy inducing agent Rapamycin has been shown to attenuate intestinal I/R induced NLRP3 inflammasome activity, thus ameliorating inflammatory responses during the course of intestinal I/R injury (21). **Table 1** summarizes the role of NLRP3 in I/R Injury in liver and intestine.

**Table 1:** Role of NLRP3 in Gastrointestinal I/R Injury.

Disease	Supplementation	Human/animal study	Cell line	NLRP3 expression	Target/signaling pathway	Observation	Ref
Hepatic I/R Injury	–	C56BL/6 mice	NPCs, KCs	Up	PINK1, IL-1β, TNF-α, Caspase-1/3, p62, LC3B, Cytochrome-c, LC3B-I/II	PINK1-mediated mitophagy by decreasing NLRP3 inflammasome activity could protect against hepatic I/R injury.	(14)
	–	Male C57/BL6 Mice	BMDMs	Up	STING, TNF-α, IFN-β, IL-1β/6/18, MCP-1, CXCL-10, Caspase-1	Aging through enhancement of STING-mediated NLRP3 activation in macrophages could aggravate liver I/R injury.	(15)
	–	Male C57 Mice	Macrophage, BMDMs	–	ATP6V0D2, p62, ASC, TNF-α, IL-1β/6/10/18, LC3-I/II, Caspase-1,	Inhibition of ATP6V0D2 *via* impairing Notch1/Hes1 signaling by promoting NLRP3 activation could aggravate liver I/R injury.	(16)
	–	Male C57BL/6J Mice	RAW 264.7	Up	SET8, MARK4, IL-1β/18, Caspase-1	SET8 through suppression of MARK4/NLRP3 inflammasome pathway could mitigate hepatic I/R injury.	(17)
	–	C57BL/6J Mice	Hepatocyte	Up	ALT, AST, ASC, IL-1β/6, TNF-α, INF-g, Ccl2, CXCR1, CXCR2, LDH	NLRP3 could regulate neutrophil and chemokine-mediated functions and contribute to hepatic I/R injury, independently of the inflammasome.	(18)
	Morin (MRN)	Male SD Rats; treated with 50 and 100 mg/kg MRN, orally, daily, for 10 days	–	Up	Nrf2, TLR4, TNF-α, IL-1β/6, MDA, MPO, TAC, Bax, Caspase-3	Morin *via* downregulating NLRP3 could alleviate hepatic I/R injury.	(22)
		Male C57BL/6J Mice	RAW 264.7	Up	CMPK2, AIM2, IL-18, IL-1β, TNF-α, Caspase-1, ALT, AST	CMPK2 *via* the NLRP3 signaling pathway could accelerate liver I/R.	(23)
	Octreotide (OTC), Melatonin (MLT)	Male Albino Rats; treated with 50 and 75 μg/kg, IP, SC, 0.5 h before the beginning of ischemia surgery, MLT 10 mg/kg prior to ischemia and again directly prior to reperfusion	–	Up	TLR-4/6, NF-κB p65, Bcl-2, Bax, Cytochrome-c, Caspase-1/9, HMGB-1	OTC and MLT through inhibition of TLR4/NF-κB/NLRP3 pathway could alleviate inflammasome-induced pyroptosis in hepatic I/R injury.	(24)
	Fisetin	Male C57BL/6J Mice; treated with 5, 10, and 20 mg/kg Fisetin, IP, 0.5 h before portal and artery hepatic occlusion	RAW264.7; 2.5, 5, 10 μmol/L fisetin during H/R	Up	GSK-3β, AMPK, TNF-α, IL-1β/18, Caspase-1, ALT, AST	Fisetin by regulating GSK-3β/AMPK/NLRP3 inflammasome pathway could mitigate hepatic I/R.	(25)
Intestinal I/R Injury	–	C57BL/6J Mice, 6 intestinal ischemia patients and 6 healthy control group	LVECs	Up	IL-1β, IL-18, IL-6, TNF-α, Caspase-1/3, E-cadherin	Activation of NLRP3 inflammasome in lung endothelial cells could contribute to intestinal I/R induced acute lung injury.	(19)
	Metformin	C57BL/6 mice; treated with 20 and 40 mg/kg metformin, IP, at the beginning of reperfusion, then applied an optimum I/R injury model	Caco-2; 1-2 mM for 30 min	Up	TXNIP, GSDMD, I-FABP, TER, ZO-1, Occludin, LDH, IL-1β/6, TNF-α	Metformin *via* the TXNIP-NLRP3-GSDMD pathway could protect against intestinal I/R injury and cell pyroptosis.	(20)
	Rapamycin (RAP), Chloroquine (CQ)	Male C57BL/6 Mice; received 3 mg/kg RAP and 60 mg/kg CQ, IP, 1 h prior to ischemia	Caco-2; 20 µmol/L CQ	Up	TNF-α, IL-6, IL-1β, Caspase-1, ASC, p62, LC3-I/II, Beclin-1	Autophagy induction *via* inhibiting NLRP3 inflammasome activation could ameliorate inflammatory responses in intestinal I/R injury.	(21)

## Neurovascular I/R injury

The role of NLRP3 has also been investigated in neurovascular I/R injury. An experiment in animal models of cerebral I/R induced by transient occlusion of middle cerebral artery and subsequent reperfusion surgery has shown down-regulation of SPATA2 expression in these models. SPATA2 has been shown to be co-localized with CYLD in neurons. Down-regulation of Spata2 has led to increased microglia, up-regulation of Tnfa, Il-1β, and Il-18, and elevation of the infarct size. Moreover, Spata2 knockdown has enhanced activity of P38MAPK, NLRP3 inflammasome and NF-κB signals (26). Another study has shown prompt activation of NLRP3 inflammasome in microglia following cerebral I/R injury onset and subsequent expression of this protein in neurons and microvascular endothelial cells afterwards. Besides, mitochondrial dysfunction has been reported to participate in activation of NLRP3 inflammasome in microglia. Thus, mitochondrial protectors could block NLRP3 inflammasome activity in art models of cerebral I/R. Taken together, NLRP3 inflammasome is activated in a cell type-dependent manner at different phases of cerebral I/R injury (27). Meanwhile, Nrf2 could inhibit activation of NLRP3 inflammasomes *via* regulating Trx1/TXNIP complex in cerebral I/R injury (28).

NLRP3 could also affect pathologic processes in acute cerebral infarction. In fact, ecosapentaenoic acid exerts its protective effects against acute cerebral infarction-associated inflammatory responses *via* suppression of activation of NLRP3 inflammasome (29). Another study has shown the impact of IMM-H004 on focal cerebral ischemia is exerted through modulation of CKLF1/CCR4- mediated NLRP3 inflammasome activation (30). Moreover, injection of IVIg could suppress NLRP1 and NLRP3 inflammasome-mediated death of neurons in cerebral I/R (31). Another study has shown down-regulation of low-density lipoprotein receptor (LDLR) expression following cerebral I/R injury. Notably, knockout of this gene in animal models has led to enhancement of caspase-1-dependent cleavage of GSDMD leading to severe pyroptosis of neurons. Mechanistically, defects in LDLR participate in the disproportionate NLRP3-facilitated maturation and release of IL-1β and IL-18 during ischemia which aggravates neurological defect and long-term cognitive function. Obstruction of NLRP3 has stunted pyroptosis of neurons in Ldlr-/- mice and cultured Ldlr-/- neurons following experimental stroke. Taken together, LDLR can modulate NLRP3-mediated pyroptosis of neurons and inflammatory responses in these cells after ischemic stroke (32). Similarly, defects in Uncoupling Protein 2 have been shown to enhances activity of NLRP3 inflammasome after hyperglycemia-associated exacerbation of cerebral I/R damage (33). Both chemical and siRNA-mediated inhibition of GSK-3β could improve neurological scores, decrease size of cerebral infarct, and reduce levels of NLRP3 inflammasome, cleaved-caspase-1, IL-1β, and IL-18. In fact, inhibition of GSK-3β activation could enhance autophagic activity (34). **Table 2** shows the role of NLRP3 in neurovascular I/R Injuries.

**Table 2:** Role of NLRP3 in Neurovascular I/R Injury.

Disease	Supplementation	Human/animal study	Cell line	NLRP3 expression	Target/signaling pathway	Observation	Ref
Cerebral I/R injury	–	Male SD Rats	–	Up	SPATA2, NF-κβ, MAPK, YNF-α, IL-1β/18, p65, p38	SPATA2 knockdown *via* NLRP3 inflammasome activation and NF-κB/P38MAPK signaling could exacerbate brain inflammation.	(26)
	–	Male SD Rats	PMC, BV-2, PC12, bEnd3	Up	Caspase-1, ASC, IL-1β/18	Mitochondrial dysfunction could induce NLRP3 inflammasome activation during cerebral I/R injury.	(27)
	–	Male SD Rats	-	Up	Nrf2, Trx1, TXNIP, IL-1β/18, Caspase-1	Nrf2 *via* regulating Trx1/TXNIP complex could inhibit NLRP3 inflammasome activation.	(28)
	CY-09	Male C57BL/6 Mice; treated with 40 mg/kg CY-09, IP, 1 h before MCAO s	Neuron; 10 μM CY-09, for 0.5 h before OGD	Up	LDLR, IL-1β/18, GSDMD, ASC, Caspase-1, p65	LDLR regulates NLRP3-mediated pyroptosis of neurons following cerebral I/R injury.	(32)
	–	Male C57BL/6 Mice	HT22	–	UCP2, ASC, SOD2, IL-1β/18, MDA, Caspase-1, TXNIP	UCP2 could enhance NLRP3 activation following HG-induced exacerbation of cerebral I/R injury.	(33)
	–	Male SD Rats	-	Up	GSK-3β, Caspase-1, IL-1β, IL-18, p62 LC3B-I/II	Inhibition of GSK-3β through suppression of NLRP3 activation *via* autophagy could alleviate cerebral I/R injury.	(34)
	–	Human; (n=15) blood samples from patients and (n=15) healthy control group	HMC3, HMO6	–	CHRFAM7A, TNF-α, Caspase-1, IL-1β, IL-6/18, iNOS, Arg1	Overexpression of CHRFAM7A *via* inhibiting microglia pyroptosis could attenuate cerebral I/R injury.	(35)
	Salvianolic Acids (SA)	Male SD Rats; treated with 10 mg/kg SA, IP, after MACO, treated with the same dose every 24 h until the day before rats sacrificing	P0–P2, primary cortical neuron; 50 μg/mL SAFI for 24 h before OGD, and then 50 μg/mL SAFI	Up	LDH, LDH, ASC, IL-1β, Caspase-1, GSDMD	SA by converting M1/M2 phenotypes and hindering NLRP3/pyroptosis axis could alleviate injury in microglia.	(36)
	Meisoindigo (MEI)	C57BL/6J Mice; treated with 2, 4, 8, 12 mg/kg MEI, IP, before MCAO and 2 h after reperfusion	HT-22, BV2; (10-150 mM MEI) at the beginning of OGD	Up	TLR4, p65, NF-κB, IL-1β, IL-18, AQP4, ASC, Arg-1, TNF-α, Caspase-1	MEI by impeding NLRP3 activation and regulating polarization of microglia/macrophage could protect against focal cerebral I/R injury.	(37)
	MCC950	Male Wistar Rats; treated with 3 mg/kg MCC950, *via* tail vein injection, 1 h, and 3 h after reperfusion	HT22, BMVEC; 100 nM MCC950	Up	AQP4, GFAP, IL-1β	NLRP3 inflammasome inhibition by MCC950 could improve diabetes-mediated cognitive impairment.	(38)
	–	Male C57BL/6J Mice	–	Up	IL-1β, Caspase-1	Inhibition of NLRP3 inflammasome could ameliorate cerebral I/R injury in diabetic mice.	(39)
	Acacetin	Male C57BL/6 Mice; treated with 25 mg/kg acacetin after MCAO for 1 h	–	Up	TLR4, NF-κβ, p65, Caspase-1, IL-1β, TNF-α, IL-6	Acacetin via the NLRP3 signaling could protect against cerebral I/R injury.	(40)
	Tetrandrine (Tet)	Male C57BL/6J Mice; treated with 30 mg/kg Tet, IP, daily, for 7 days and 30 min before and after MCAO	–	Up	Sirt-1, IL-1β/18, Caspase-1	Tet *via* Sirt-1 could alleviate cerebral I/R injury by suppressing the activation of NLRP3 inflammasome.	(41)
	Bakuchiol (BAK), Brusatol (Bru)	Male C57BL/6 Mice; treatment with 2.5 and 5 mg/kg BAK per day for 5 days	BV-2; 200 nM Bru for 6 h, then incubated with 2.5-5 μM BAK for 2 h, followed by OGD/R induction	Up	Nrf2, ASC, HO-1 Caspase-1/3, IL-1β/18, Histone H3	BAK by modulating NLRP3 inflammasome and Nrf2 signaling could ameliorate cerebral I/R.	(42)
	Qingnao Dripping Pills (QNDP)	Male SD Rats; treated with 0.15 g/kg QNDP, orally, 2 h after MCAO	SH-SY5Y; 5µg/mL during OGD	Up	Bad, Bcl-XL, NF-κβ, Caspase-1/3, IL-1β, IL-18, ASC	QNDP *via* inhibiting NLRP3 could protect against cerebral I/R injury.	(43)
	Tomentosin (TOM)	Male SD Rats; treated with 25 and 50 mg/kg TOM for consecutive 7 days	SH-SY5Y; 10 μg, 20 μg, 30 μg TOM, for 24 h	Up	IL-1β, TNF-α, IL-4/6/10, VEGF, Caspase-1, TLR4, LDH, Catalase, Glutathione peroxidase, Glutathione, Lipid peroxidation, Acetylcholine	TOM *via* TLR4/NLRP3 signaling could inhibit cerebral I/R injury.	(44)
	Qingkailing (QKL)	Male SD Rats; 3 ml/kg QKL, IP, injected immediately after model establishment, followed by 4 h, and once every 12 h post-treatment	–	Up	AMPK, TNF-α, IL-4/6/10, IL-1β, MDA, SOD, ASC, Caspase-1	QKL *via* modulating AMPK/NLRP3 signaling could ameliorate cerebral I/R injury.	(45)
	PAP-1	Male SD Rats; treated with 40 mg/kg PAP-1, IP, after MCAO and reperfusion operation. Also, treated with the same dose of PAP-1 every 12 h until the day before rats sacrificing	P0–P2; 50 nM PAP-1, for 24 h	Up	IL-1β, M1, M2, Caspase-1	Kv1.3 channel blockade by reformatting M1/M2 phenotypes and modulating NLRP3 inflammasome activation could alleviate cerebral I/R in microglia.	(46)
	Adiponectin (APN)	Male C57BL/6 J Mice; treated with 2, 20, and 25 μg/g, IP, immediately after MACO	Primary astrocytes; 50 μM APN	Up	AMPK, GSK-3β, Caspase-1/3, p20, IL-1β/18, ASC, Bcl-2, Bax, Nrf2	APN peptide by regulating AMPK/GSK-3β could alleviate oxidative stress and NLRP3 inflammasome activation.	(47)
	Procyanidins	Male SD Rats; treated with 20-80 mg/Kg injected 1 h before occlusion	BV2, 0.01–100 μM	Up	TLR4, NF-κB, Bcl-2, Bax, p38, Caspase-1, IL-1β	Procyanidins by inhibiting the TLR4-NLRP3 inflammasome pathway could exhibit neuroprotective activities against cerebral I/R injury.	(48)
	Sulforaphane (SFN), Genipin, MCC950	Male C57Bl/6N Mice; treated with SFN, Genipin, MCC950 (25, 2, 50 mg/kg), *via* IP injection either directly before occluding the MCA or after the 1 h of tMCAO	–	Up	NLRP1a/b, NLRC4, AIM2, IL-1β/18, Caspase-1	Early blockade of NLRP3 by stabilizing the blood-brain barrier and mitigating inflammation could protect from I/R injury.	(49)
	Cepharanthine (CEP)	Male C57/BL6 mice; treated with 10 or 20 mg/kg CEP 0.5 h before MCAO and supplemented 12 h after MCAO *via* IP injection	BV-2; CEP (0.25, 0.5, 1, 2.5 μg/mL) for 30 min	Up	ALOX15, Caspase-1, IL-1β/18, SOD, MDA	CEP by reducing oxidative stress *via* inhibiting 12/15-LOX signals and NLRP3 inflammasome-induced inflammation could attenuate cerebral I/R injury.	(50)
	Electroacupuncture(EA)	Male SD Rats; stimulated with the frequency of 2/15 Hz and an intensity of 1 mA for 30 min EA, for 5 days	–	Up	α7nAChR, Caspase-1, GSDMD, IL-1β/18, TGF-β1, TNF-α	EA *via* suppression of NLRP3 inflammasome could attenuate cerebral I/R neuroinflammation in stroke rats.	(51)
	Butyphthalide (NBP), CD21	C57BL/6J mice; treated with 2.5, 5, and 10 mg/kg IV at 1 min, 24 h, and 48 h after reperfusion	–	Up	Caspase-1, IL-1β, IL-6, TNF-α, TLR4, NF-κB	Phthalide derivative CD21 *via* inhibiting NLRP3 could ameliorate cerebral I/R injury.	(52)
	Hispidulin (His)	Male SD Rats; treated with 40-80 mg/kg His once daily for 3 days following I/R, IP injection	Astrocytes; His (5-10 μM) for 2 h	Up	Caspase-1, IL-18, IL-1β, AMPK, GSK-3β, ASC	Hispidulin *via* suppressing NLRP3-mediated pyroptosis could exhibit neuroprotective activities against cerebral I/R injury	(53)
	Idebenone	Male SD Rats; treated with 100 mg/kg Idebenone, IP	Primary microglial cells, BV2, PC12; (Idebenone: 0.2-2.0 μM) added after reoxygenation	–	Caspase-1, IL-1β/18, ROS, NQO1/2, NOX2	Idebenone *via* dampening NLRP3 inflammasome activity could attenuate cerebral I/R injury.	(54)
	D-Carvone	Male Wistar Rats; treated with 10 and 20 mg/kg D-Carvone (IP) 15 min before reperfusion, daily for 15 days	–	Up	TLR3/4, TNF-α, IL-1β, Caspase-1, IL-4/6/10, VEGF, ASC	D-Carvone *via* downregulating TLR4/NLRP3 pathway could inhibit cerebral I/R injury.	(55)
Cerebral Ischemic Stroke (CIS)	Ki20227	Male C57BL/6 Mice; pretreatment with Ki20227 (0.002 mg/kg), daily, for 7 days, orally, then mice administrated once for the next 24 h after ischemia induction	–	Up	CSF1R, TNF-α, IL-10, Arg-1, iNOS, NF-κB, Caspase-1	Downregulation of CSF1R *via* inhibiting microglia M1 polarization and NLRP3 could alleviate cerebral ischemic stroke.	(56)
	Genistein (Gen)	Female C57BL/6J Mice; treatment with 10 mg/kg Gen, IP, for 14 days, prior to MCAO	HT22, N9, primary mouse microglia, received Gen for 24 h	Up	Caspase-1, IBA-1, IL-1β, Il-18, TNF-α, LDH	Gen by inhibiting the NLRP3 in could attenuate acute CIS.	(57)
Acute Cerebral Infarction (ACI)	Eicosapentaenoic (EPA)	C57BL/6 Mice; treated with 0, 10, 20, and 30 mg/kg EPA, orally once daily for 2 weeks before experiments began	BV-2; (0-30 μmol EPA)	–	Caspase-1/3, IL-1β, ASC, IL-18, MCP-1, TNF-α	EPA *via* inhibiting NLRP3 inflammasome activation could prevent inflammation induced by ACI.	(29)
Focal Cerebral Ischemia (FCI)	IMM-H004	Male SD Rats; received 2.5, 5, 10 mg/kg IMM-H004	–	–	CKLF1, CCR4, IL-1β/18, TNF-α, ASC, Caspase-1, LDH	IMM-H004 *via* modulation of CKLF1/CCR4-mediated NLRP3 inflammasome activation could mitigate FCI.	(30)
	Intravenous Immunoglobulin (IVIg)	Male C57BL/6J Mice; 1 g/kg IVIg, by infusion into the femoral vein, after reperfusion (3 h); Human Brain Tissues	PCN	Up	NLRP1, ASC, XIAP, caspase-1/3/11, IL-1β/18	Injection of IVIg could suppress NLRP1 and NLRP3 inflammasome-mediated death of neurons in cerebral I/R.	(31)
Hypoxia Ischemic Stroke	YC-1	Male SD Rats; treated with 5 mg/kg YC-1 *via* IP injection 2 hours before MCAO	–	Up	HIF-1α, Caspase-1, IL-1β, IL-18, MPO	YC-1 *via* downregulating HIF-1α could inhibit NLRP3 inflammasome-dependent pyroptosis and apoptosis.	(58)
Hemorrhagic Transformation (HT)	Melatonin	Male SD Rats; treated with 15, 50, and 150 mg/kg melatonin IP injected to rats at 2 h after MCAO	–	Up	ROS, IL-1β, Caspase-1,	Melatonin *via* suppressing ROS-induced NLRP3 activation after cerebral ischemia could ameliorate HT in hyperglycemic rats.	(59)
	Peroxynitrite (PN)	Male SD Rats; treated with 3 mg/kg uric acid (a peroxynitrite scavenger) and 16 mg/kg FeTmPyP (a representative peroxynitrite decomposition catalyst), IV, upon reperfusion	b.End3, PC12; (20, 40 μM PN, for 2 h)	Up	iNOS, p47phox, MMP-2/3/9, ASC Caspase-1, IL-1β	PN *via* activating NLRP3 inflammasome could contribute to HT and poor outcomes in ischemic stroke.	(60)
Ischemia Brain Injury (IBI)	Panax Ginseng and Angelica (CPA), ginsenoside Rd (Rd) and Z-ligustilide (LIG)	Male SD Rats; treated with (4.5 and 9 g/kg CPA; orally, once daily, for 3 days before MCAO and for 7 days following MCAO	BV-2; Rd (0.1, 1.0, 10 μmol/l) and LIG (1, 2.5, 10 μmol/l) alone or in combination, for 2 h, and then exposed to OGD/R	Up	Caspase-1, IL-1β, GSMD, GSMD-NT, LDH, DRP1,	The combination of CPA through inhibiting NLRP3 activation and microglial pyroptosis could alleviate IBI.	(61)
	–	SD Rats	–	Up	Caspase-1, IL-1β	Activation of NLRP3/Caspase-1/IL-1β signaling could enhance after IBI.	(62)
	Progesterone (PROG)	Male SD Rats; 8 mg/kg PROG, IP, injected 2 h post-ischemia followed by S.C injection at 6 h, and once every 24 h post-injury for 5 days	–	Up	HMGB1, TLR4, ASC, Caspase-1, IL-1β, IkBa, LC3-I/II, LC3	PROG *via* enhancing autophagy following IBI could attenuate stress-induced NLRP3 activation.	(63)
Spinal Cord I/R Injury	–	Male SD Rats	PRSCA	Up	NF-κβ, GFAP, p65, p20, HSPA8, ASC, Caspase-1, IL-1β/18	Inhibition of HSPA8 via astrocyte NF-κβ/NLRP3 inflammasome axis could attenuate spinal cord I/R injury.	(64)
Retinal I/R Injury	Puerarin	Male SD Rats; treated with 25, 50 100 mg/kg puerarin at 1h, 24h, and 48h after I/R	RGCs; (100 μM puerarin)	–	TLR4, ASC, IL-18, IL-1β, MyD88, TRAF-6, Caspase-1	Puerarin through suppression of the activation of TLR4/NLRP3 inflammasome could ameliorate retinal ganglion cell damage induced by retinal I/R.	(65)
	Sulforaphane (SFN)	Female SD Rats; treated with 5, 10, and 20 mg/kg SFN, orally started 1-week before acute glaucoma surgery	RGCs	Up	TNF-α, IL-1β, MHC-II, ASC, Caspase-3	SFN by suppressing NLRP3 inflammasome could alleviate retinal ganglion cell death.	(66)
	–	Male Brown Norway Rat	–	Up	TLR2/4, MyD88, TRAF6, NF-κβ, NLRP1, ASC, Caspase-1/3, IL-1β/18	Retinal I/R could mediate by TLR4 activation of NLRP3 inflammasome.	(65)

## Myocardial I/R injury

**Figure 3:**
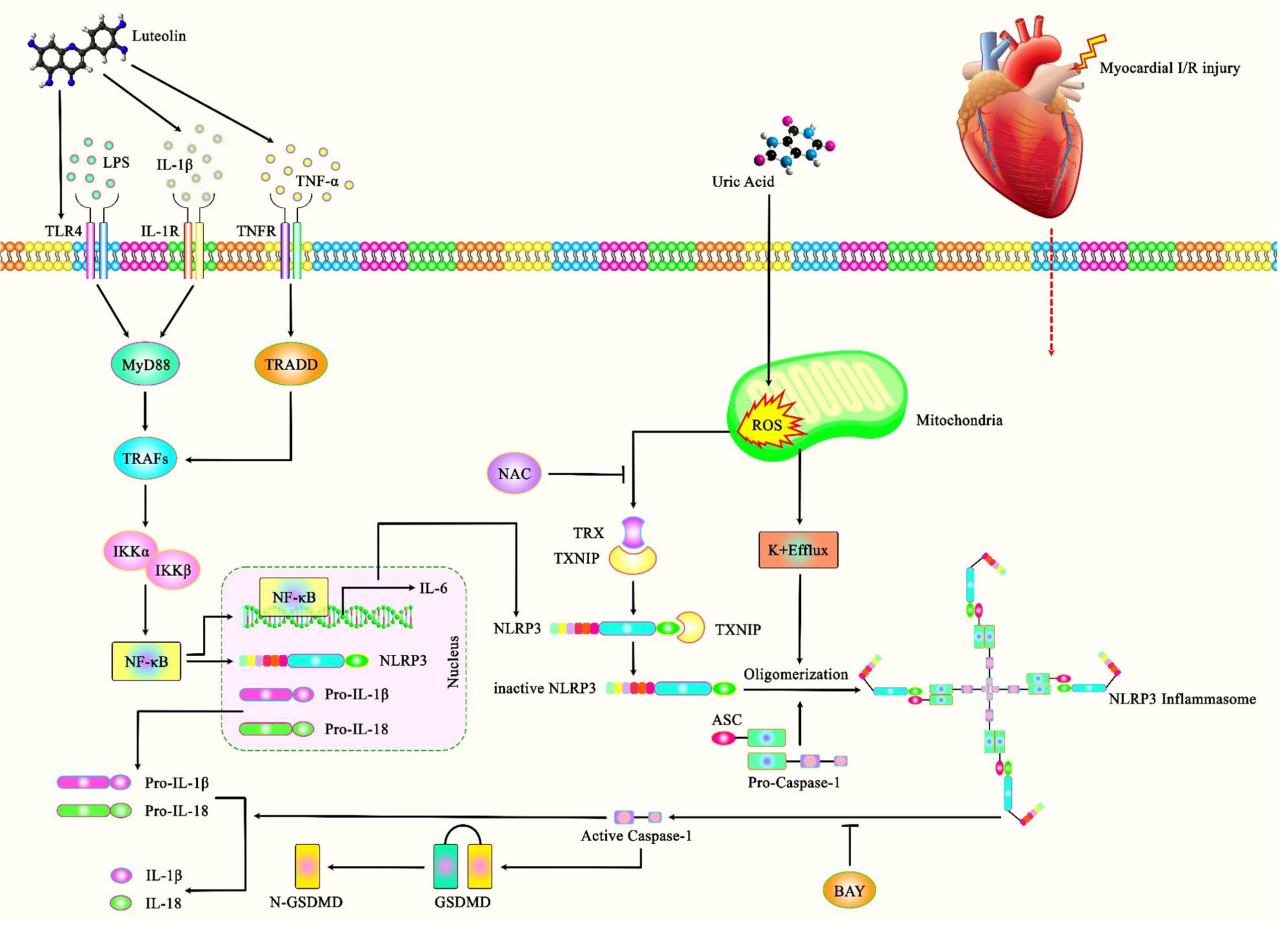
A schematic illustration of the role of NLRP3 Inflammasome and its central role in the myocardial I/R injury. Accumulating evidence has illustrated that Luteolin could have a key role in protecting against myocardial I/R injury as opposed to Uric acid that could aggravate this injury through ROS/NLRP3 pyroptosis pathway. It has been reported that Luteolin could protect against myocardial I/R injury through TLR4/NF-kB/NLRP3 inflammasome cascade by downregulating the expressions of NLRP3, ASC, caspase-1, TLR4, MyD88 and the phosphorylations of IKKα, IKKβ, IκBα, and NF-κB (71).

NLRP3 inflammasome-associated pyroptosis is also involved in myocardial I/R injury. IP3R1 protein that regulates release of Ca2+ from endoplasmic reticulum has been shown to regulate pyroptosis through the NLRP3/Caspase-1 axis in myocardial I/R injury (67). Cardiac I/R injury can be alleviated through Calpain silencing which affects activity of the LRP3/ASC/Caspase-1 axis (68). Similarly, Formononetin has been shown to suppress the ROS-TXNIP-NLRP3 axis to ameliorate myocardial I/R injury in rats (69). In contrast, uric acid could aggravate myocardial I/R injury *via* ROS/NLRP3 pyroptosis pathway (70) (**Figure 3**).

Another study has demonstrated that diabetes aggravates myocardial I/R injury *via* influencing NLRP3 inflammasome-associated pyroptosis. Notably, suppression of inflammasome activation using BAY11-7082 has reduced the myocardial I/R injury in exposed animals. Consistently, both BAY11-7082 and the antioxidant N-acetylcysteine could reduce high glucose and hypoxia/reoxygenation-induced injuries in cardiomyocytes *in vitro*. Taken together, high glucose-induced NLRP3 inflammasome activation might depend on ROS production, and NLRP3 inflammasome-associated pyroptosis exacerbates myocardial I/R injury in diabetic animals (72). **Table 3** shows the impact of NLRP3 in myocardial I/R Injury.

**Table 3:** Impact of NLRP3 in Myocardial I/R Injury.

Disease	Supplementation	Human/animal study	Cell line	NLRP3 expression	Target/signaling pathway	Observation	Ref
Myocardial I/R Injury	–	SD Rats	H9C2	Up	IP3R1, ERP44, ASC, IL-1β/18, Caspase-1, CK-MB, GSDMD-N	IP3R1 *via* the NLRP3/Caspase-1 pathway could regulate Ca^2+^ transport and pyroptosis.	(67)
Myocardial I/R Injury	–	Male C57BL/6 Mice	-	Up	Calpain, ASC, Caspase-1, CHOP, GRP78, C/EBP	Calpain silencing *via* the LRP3/ASC/Caspase-1 axis could alleviate myocardial I/R injury in mice.	(68)
	–	Male SD Rats	H9C2	Up	CK-MB, LDH, ROS Caspase-1, IL-1β,	NLRP3 inflammasome activation could aggravate myocardial I/R in diabetic rats.	(72)
	Formononetin (FN)	Male SD Rats; treated with 10 and 30 mg/kg FN following 60 min ischemia *via* IP injection	NRCMs; 1 and 10 µM FN for 2 h	Up	TXNIP, Bcl-2, Bax, Caspase-1, ASC, TNF-α, IL-1β, IL-6	Formononetin by suppressing the ROS-TXNIP-NLRP3 pathway could ameliorate myocardial I/R injury in rats.	(69)
	Potassium Oxonate (PO)	Male Kunming Mice; treated with 300 mg/kg PO for 14 consecutive days	Cardiomyocyte, 100-400 mg/L	Up	Caspase-1/3, ASC, IL-1β	Uric acid *via* ROS/NLRP3 pyroptosis pathway could aggravate myocardial I/R injury.	(70)
	Metformin	SD Rats; treated with 50 µM metformin for 15 min besides I/R injury	NRVMs, 5 mM metformin, 30 min	–	AMPK, IL-1β, IL-18, TNF-α, Caspase-1 ACC, Bax, BCl-2, COL-I/III, CK-MB, cTnI	Metformin *via* AMPK/NLRP3 inflammasome pathway could protect against myocardial pyroptosis.	(73)
	Ethyl Pyruvate (EP)	Male SD Rats; treated with 50 mg/kg EP *via* tail vein 1h before ischemia	H9c2; 10 mM EP	Up	ASC, Caspase-1, IL-1β, NOX4, CPT1A, TXNIP, ERK, p38	EP *via* regulating ROS-related NLRP3 inflammasome activation could protect against myocardial I/R.	(74)
	M2 Macrophage-Derived Exosomes (M2-exos)	SD Rats; injected M2-exos 2–3 μg per rat *via* the caudal vein 2h before I/R procedure	NRCMs; pretreated with M2-exos	Up	miR-148a, TXNIP, TLR4, NF-κB, ASC, p65, IL-1β, IL-4/18, lκBα	M2-exos carried miR-148a through suppression of TXNIP and the TLR4/NF-κB/NLRP3 inflammasome could alleviate myocardial I/R injury.	(75)
	Scutellarin (Scu)	SD Rats; treatment with 5, 10, and 20 mg/kg Scu, IP, 15 min before vascular ligation	H9c2; 3.125, 6.25, 12.5 µg/ml Scu, 6 h before OGD	Up	CK-MB, cTnI, Myo, P62, Beclin-1, LC3BII/I, TNF-α, IL-1β, IL-18, Caspase-1, HK-1, Akt	Scu by suppressing NLRP3 inflammasome activation could protect against myocardial I/R.	(76)
	Luteolin (Lut)	Male SD Rats; treated with 40, 80, 160 mg/kg Lut, orally, for 7 days before the operation	H9c2; 5, 10, and 20 mM Lut	Up	TLR4, NF-κβ, IL-1β/18, TNF-α, MyD88, ASC, Caspase-1, CK-MB, IKKa/β, IkBα	Lut *via* targeting TLR4/NF-κβ/NLRP3 inflammasome pathway could modulate myocardial I/R injury.	(71)
	OLT1177 (Dapansutrile)	Male ICR (CD1) Mice; treated with 6, 60, 600 mg/kg OLT1177, IP, after 60, 120, 180 min reperfusion	–	–	Caspase-1	Inhibition of NLRP3 inflammasome by OLT1177 could reduce infarct bulk and preserve contractile function after I/R injury in mice.	(77)
	–	Male C57BL/6 Mice	PMN	–	TLR4, CRAMP, P2X7R, cTnI, IL-1β/6, TNF-α	Cathelicidin *via* activating TLR4 and P2X7R/NLRP3 inflammasome could aggravate myocardial I/R injury.	(44)
	Puerarin (Pue)	Male C57BL/6 mice; treated with 100 mg/kg Pue, IP, 15 min or 20 min prior to reperfusion	–	Up	SIRT1, NF-κβ, CK-MB, TNF-α, IL-1β/6/18, Caspase-1/3, Bcl-2, Bax, p65	Puerarin by inhibiting inflammation and the NLRP3 inflammasome could protect against myocardial I/R.	(78)
	Hydrogen gas (HG)	Male SD Rats; inhaled 2% concentration HG	–	Up	ROS, MDA, 8-OHdG, Caspase-1, p20, ASC, IL-1β, cTnI	HG inhalation by the inhibition of oxidative stress and NLRP3-mediated pyroptosis could alleviate myocardial I/R injury.	(79)
	Biochanin A (BCA)	Male SD Rats; treated with 12.5, 25, and 50 mg/kg BCA, IP, every day for 7 days before the operation	–	Up	TLR4, NF-κB, CK-MB, AST, IL-1β, IL-6/18, TNF-α, MyD88, lκBα, ASC, Caspase-1	BCA *via* the TLR4/NF-κB/NLRP3 signaling could attenuate myocardial I/R injury.	(80)
	SRT1720	SIRT1^flox/flox^, CreER^T2^, and wild-type C57BL/6 Mice; treated with 30 μg/g SRT1720, IP, before surgery	–	Up	IL-1β/18, ROS, PI3K, AKT, PDH, AMPK, ACC, SIRT1, PDK, PTEN, CPT-1, PDHK1, PDH E1α, AMPKα	SIRT1 agonist *via* pyruvate dehydrogenase could modulate cardiac NLRP3 inflammasome during ischemia and reperfusion.	(81)
Acute Myocardial Infarction (AMI)	IL-17A	Wild-type C57BL/6 Mice; treated with 0, 20, and 50 ng/mL IL-17A for 12 h	–	–	AMPKα, p38MAPK, ERK1/2, IL-1β, ACC, p20, JNK	IL-17A *via* AMPKα/p38MAPK/ERK1/2 signaling by activating NLRP3 inflammasome could contribute to myocardial ischemic injury in mice.	(82)
	Electroacupuncture (EA)	Male C57BL/6 Mice; stimulated with 2/15 Hz with an intensity level of 2 mA for 20 min, daily, for 3 days	–	Up	IL-1β, Caspase-1	EA preconditioning *via* inhibiting NLRP3 inflammasome activation could attenuate AMI in mice.	(83)
Ischemic Heart Disease (IHD)	Resveratrol (RSV)	Male C57BL/6J Mice; treated with 320 mg/kg RSV, orally, at 8 a.m and 5 p.m, 1 week before MI surgery	NRCMs, CFs, Macrophage, -	Up	P16/19/20/53, SIRT1, MMP-2/9, Caspase-1/3, IL-1β, IL-6, TNF-α, Bax	RSV *via* targeting NLRP3 inflammasome activation could inhibit ischemia-induced myocardial senescence signals.	(84)
Ischemic Stroke	Vinpocetine (Vinp)	Male C57BL/6 Mice; treated with 5 and 10 mg/kg Vinp, IP, 1 h after reperfusion	–	Up	IL-1β, IL-18, NF-kB, ASC, Caspase-1,	Vinp *via* inhibiting NLRP3 inflammasome expression could attenuate ischemic stroke in mice.	(85)

## Other types of I/R injury

The role of NLRP3 has also been assessed in limb, renal and testicular I/R injuries (**Table 4**). For instance, an experiment in rats has shown that hydrogen-rich saline decreases acute limb I/R-induced lung injury through decreasing levels of chemerin and NLRP3 (86).

**Table 4:** Role of NLRP3 in other types of I/R Injury.

**Disease**	**Supplementation**	**Human/animal study**	**Cell line**	**NLRP3 expression**	**Target/signaling pathway**	**Observation**	**Ref**
Limb I/R Injury (LI/R)	Hydrogen-Rich Saline (HRS)	Male Wistar Rats; treated with 2.5 and 10 mL/kg HRS, IP, immediately after the femoral artery occlusion		Up	CHEMERIN, IL-6, TNF-α, MDA, SOD	HRS decreases acute limb I/R-induced lung injury through decreasing levels of chemerin and NLRP3.	(86)
Femoral Artery Ligation (FAL)	–	Male C57BL/6 Mice	–	Up	TLR4, IL-1β, Caspase-1	The platelet NLRP3 inflammasome could promote platelet aggregation.	(87)
Renal I/R Injury	–	Male C57BL/6J Mice, Human Kidney Biopsy Specimens		Up	–	NLRP3 is overexpressed in chronic kidney disease after I/R induced-acute renal injury.	(88)
Testicular I/R Injury	–	Male C57BL/6J Mice, KO Mice	–	–	Caspase-1/3, IL-1β/18	NLRP3 inflammasome participates in the impairment of spermatogenesis following testicular I/R.	(89)

Moreover, TLR4-associated increase in the activity of the platelet NLRP3 inflammasome has been reported to promote aggregation of platelets in a mice model of hindlimb ischemia (87). Similarly, I/R induced-acute kidney injury has been shown to be associated with over-expression of NLRP3 is overexpressed in chronic kidney disease (88). NLRP3 inflammasome is also implicated in the tissue injury and impairment of spermatogenesis caused by testicular I/R (89). Specific inhibitors of NLRP3 inflammasome, namely BAY 11-7082 (90) and Brilliant Blue G (BBG) (91) have been found to suppress effects of NLRP3 in an animal model of testicular I/R (89). Both agents could significantly reduce expressions of IL-1β and IL-18, diminish caspase-1 and caspase-3 levels and preserve spermatogenesis, representing a selective decrease in the activity of NLRP3 inflammasome (89). Moreover, NLRP3 knock-out mice has responded to I/R injury with a diminished level of induction of inflammatory and apoptosis cascade compared with wildtype animals. Thus, NLRP3 might be an appropriate target for new drugs for treatment of I/R injury after testicular torsion (92).

## Discussion

NLRP3 is an essential element in the inflammasome whose activation by tissue injuries or pathogens leads to cleavage of caspase-1 by an autocatalytic process and release of inflammatory factors IL-1β and IL-18 (10). Thus, abnormal function of NLRP3 has been associated with development of several immune-related disorders. Consistently, NLRP3 targeting has been suggested as an interesting method for design of therapeutic modalities for management of NLRP3 inflammasome-related disorders (93). Several *in vitro* and animal studies have assessed the role of NLRP3 in gastrointestinal, neurovascular and cardiac I/R injuries. The results of these studies indicate critical role of this protein in induction of I/R injuries in different tissues. Since I/R injuries are associated with morbidity and mortality, targeting NLRP3 is a possible strategy for reduction of disease burden. In fact, inhibition of NLRP3 inflammasome activity can ameliorate inflammatory responses in intestinal or hepatic I/R injury. NLRP3 inflammasome can also aggravate pathologic events in ischemic brain injury, spinal cord injury and retinal injury. Thus, modulation of this cellular mechanism can be an effective strategy for treatment of a wide variety of disorders, particularly those associated with aging.

A number of known protective agents against cerebral injuries such as salvianolic acids, meisoindigo, acacetin, tetrandrine, bakuchiol, tomentosin and qingkailing have been shown to exert their effects through modulation of expression of NLRP3. Similarly, a number of substances such as formononetin, metformin, ethyl pyruvate, scutellarin, luteolin, OLT1177 (Dapansutrile), puerarin and biochanin have been found to protect against myocardial I/R injury *via* suppression of NLRP3. Thus, targeting NLRP3 is a promising strategy for management of different types of I/R injury.

Previous studies have reported association between NLRP3 genetic variants and risk of inflammatory conditions such as rheumatoid arthritis (94) and inflammatory bowel diseases (95). However, the exact impacts of these variants on I/R injuries have not been identified yet. Identification of the role of these variants in induction of I/R injuries would facilitate recognition of individuals being at risk of myocardial/cerebral injuries.

Future studies are needed to find novel substances for amelioration of NLRP3-mediated I/R injuries. Moreover, the functional interactions between NLRP3 and other molecules that contribute in the I/R injuries should be identified to further design more effective therapies for this kind of tissue injuries.

## Author contributions

SG-F wrote the draft and revised it. MT designed and supervised the study. HSH, YP, BMH, YH, AA, and AE collected the data and designed the figures and tables. All authors contributed to the article and approved the submitted version.

## Conflict of interest

The authors declare that the research was conducted in the absence of any commercial or financial relationships that could be construed as a potential conflict of interest.

## Publisher’s note

All claims expressed in this article are solely those of the authors and do not necessarily represent those of their affiliated organizations, or those of the publisher, the editors and the reviewers. Any product that may be evaluated in this article, or claim that may be made by its manufacturer, is not guaranteed or endorsed by the publisher.

## Abbreviations

tMCAO, transient middle cerebral artery occlusion; MCA, middle cerebral artery; CIS, Cerebral ischemic stroke; CSF1R, colony-stimulating factor 1 receptor; KCs, Kupffer cells; RGCs, retinal ganglion cells; LVECs, lung vascular endothelial cells; BMDMs, Bone marrow-derived macrophages; NRCMs, neonatal rat cardiomyocytes; NRVMs, Neonatal rat ventricle myocytes; CFs, cardiac fibroblasts; PRSCA, Primary rat spinal cord astrocytes; CMPK2, Cytidine monophosphate kinase 2; AIM2, absent in melanoma 2; MDA, Malondialdehyde; MPO, myeloperoxidase; TAC, total antioxidant capacity; AMH, anti-Müllerian hormone; TER, transepithelial electrical resistance; I-FABP), intestinal fatty binding protein; CAT, Catalase; Sirt-1, Sirtuin-1; H/R, Hypoxia/reoxygenation; OGD, oxygen and glucose deprivation; IP3R1, Intracellular ion channel inositol 1,4,5-triphosphate receptor; LDLR, Low-density lipoprotein receptor; BMVECs, brain microvascular endothelial cells; PMNs, polymorphonuclear leukocytes; HSPA8, heat shock protein family A; CHOP, C/EBP homologous protein; CCR4, chemokine receptor type 4; MARK4, microtubule affinity-regulating kinase 4; Drp1, dynamin-related protein 1; UCP2, uncoupling protein 2; CKLF1, Chemokine-like factor 1; PMC, Primary microglial cell; Trx1, hioredoxin1; Nrf2, Nuclear factor erythroid 2-related factor 2; TXNIP, thioredoxin interacting protein; AIM2, absent in melanoma 2; NLRC4, CARD domain containing 4; TLR, Toll-like receptor 2; TRAF6, TNF receptor-associated factor 6; PCN, Primary cortical neuron; XIAP, X-linked inhibitor of apoptosis protein; AMPK, AMP-activated protein kinase; CRAMP, antimicrobial peptide Cathelicidin.
